# Protective Effects of Fuziline on Dobutamine-Induced Heart Damage in Mice

**DOI:** 10.21470/1678-9741-2022-0251

**Published:** 2023-06-14

**Authors:** Yasemin Hacanli, Mehmet Salih Aydin, Ezhar Korkmaz Ersöz, Nazim Kankiliç, İsmail Koyuncu, Muhammet Emin Güldür, Ebru Temiz, Reşat Dikme, Kadir Eği, Yusuf Çakmak, Mahmut Padak

**Affiliations:** 1 Department of Cardiovascular Surgery, Medical School of Harran University, Şanliurfa, Turkey; 2 Departmant of Medicinal Biochemistry, Medical School of Harran University, Şanliurfa, Turkey; 3 Department of Pathology, Islamic Science and Technology University, Gaziantep, Turkey; 4 Medical Promotion and Marketing Program, Health Services Vocational School, Harran University, Şanliurfa, Turkey; 5 Perfusion Techniques Program, Health Services Vocational School, Harran University, Şanliurfa, Turkey; 6 Animal Experiment Application and Research Center (HDAM), Harran University, Şanliurfa, Turkey

## Abstract

**Introduction:**

Fuziline is one of the many antioxidants currently being tested to treat cardiac damage. In our study, histopathological and biochemical effects of fuziline were investigated in mice with dobutamine-induced heart damage in vitro.

**Methods:**

Thirty-two adult male BALB/c mice, average weight of 18-20 g, were randomly divided into four groups - Group 1 (sham, n=8), Group 2 (control, dobutamine, n=8), Group 3 (treatment 1, dobutamine + fuziline, n=8), and Group 4 (treatment 2, fuziline, n=8). Biochemical parameters and total antioxidant status (TAS), total oxidant status (TOS), and oxidative stress index (OSI) values were measured. Interleukin 1 beta (IL-1β), NLR family, pyrin domain containing protein 3 (NLRP3), 8-hydroxy-deoxyguanosine (8-OHDG), gasdermin D (GSDMD), and galectin 3 (GAL-3) levels were analyzed by enzyme-linked immunosorbent assay method, and histopathological examination of heart tissues was performed.

**Results:**

When dobutamine + fuziline and fuziline groups were compared, troponin-I (P<0.05), NLRP3 (P<0.001), GSDMD (P<0.001), 8-OHDG (P<0.001), IL-1β (P<0.001), and GAL-3 (P<0.05) were found to be statistically significant. TOS level was the highest in the dobutamine group (P<0.001) and TAS level was the highest in the fuziline group (P<0.001). OSI level was statistically significant between the groups (P<0.001). In histopathological examination, focal necrosis areas were smaller in the dobutamine + fuziline group than in the dobutamine group, and cardiac myocytes were better preserved.

**Conclusion:**

Fuziline markedly reduced cardiac damage and pyroptosis in mice with dobutamine-induced heart damage by lowering the levels of GSDMD, 8-OHDG, IL-1β, and GAL-3. It also prevented necrosis of cardiac myocytes in histopathological evaluation.

**Table:** 

Abbreviations, Acronyms & Symbols				
8-OHDG	= 8-hydroxy-deoxyguanosine		ISO	= Isoproterenol
ALT	= Alanine aminotransferase		K	= Potassium
AU	= Arbitrary units		MI	= Myocardial infarction
Cr	= Creatinine		Na	= Sodium
CVD	= Cardiovascular disease		NLRP3	= NLR family, pyrin domain containing protein 3
DNA	= Deoxyribonucleic acid		OSI	= Oxidative stress index
ECG	= Electrocardiogram		PM2.5	= Particulate matter 2.5
GAL-3	= Galectin 3		ProBNP	= Pro-brain natriuretic peptide
GSDMD	= Gasdermin D		RNS	= Reactive nitrogen species
HF	= Heart failure		ROS	= Reactive oxygen species
IL-1β	= Interleukin 1 beta		SD	= Standard deviation
IL-18	= Interleukin 18		TAS	= Total antioxidant status
IP	= Intraperitoneally		TOS	= Total oxidant status

## INTRODUCTION

Many factors play a role in the formation of cardiovascular diseases (CVDs). These include modifiable factors, such as obesity, hypertension, diabetes, etc., and non-modifiable factors, such as genetic predisposition, age, and gender^[1]^. Diseases such as heart attack, heart failure (HF), and coronary heart disease are among CVDs that may cause death^[2]^. A positive inotropic agent is recommended to maintain end-organ function and systemic perfusion in patients admitted to hospitals due to heart disease^[3]^. Agents that increase cardiac output by increasing the contractility of the heart muscle are called (positive) inotropic agents^[4]^. Dobutamine is widely used as a positive inotropic agent; it is formed from isoproterenol (ISO), and its vascular and arrhythmogenic effects are less than those of other positive inotropic agents^[5]^. Long-term use of dobutamine may initiate significant ventricular arrhythmias and cause sudden death^[6]^. In CVD models created with dobutamine, it has been observed that oxidative stress increases generally and dobutamine creates an immunosuppression on the immune system.

Oxidative stress occurs with the intense presence of free radicals and oxidants in cells^[7]^. These radicals are reactive oxygen species (ROS) and reactive nitrogen species (RNS)^[8]^. Nitrosative and oxidative stress are manifested by an increase in RNS and ROS synthesis or a decrease in the antioxidant system, respectively. The reason for this is the deterioration of the oxidant-antioxidant balance against antioxidants because, under normal conditions, ROS is removed from the environment thanks to enzymatic and non-enzymatic antioxidants and tissues are protected from oxidation^[9]^.

ROS found in high concentration in cells can cause ferroptosis, apoptosis, and pyroptosis. Along with these events, the export of inflammatory cytokines and the synthesis of excessive amounts of ROS can initiate further cell death. Because the pathways that carry out cell death are interconnected^[10]^, increasing the concentration of free radicals in the cell also plays a role in the formation of CVD and various chronic diseases^[11]^.

Many drugs and antioxidants are being tried for the treatment of cardiac injury. In recent years, different approaches have been tested to prevent cardiac injury. In the studies conducted by Erdemli et al.^[12]^, it was revealed that powerful antioxidants are effective in reducing oxidative stress, improving the damage to the heart tissue caused by inflammatory disorders^[13]^ and reducing the rate of hepatotoxicity^[14]^.

Studies suggest that phenolic compounds with antioxidant effect prevent disorders^[15]^. Some studies reveal that phenolic compound support is effective in reducing or preventing the emergence of disorders such as diabetes and CVD associated with oxidative stress^[16]^. The largest group of phenolic compounds are flavonoids, which occupy the first place in studies^[17]^. Fuzi (or Radix Aconiti Lateralis Preparata), described as a Chinese herb and possessing antioxidant properties, is the derived form of *Aconitum carmichaelii Debx*^[18]^. Fuzi has 122 chemical components, especially flavonoids, alkaloids, fatty acids, and saponins^[19]^. Scientific studies on Fuzi have also been intensively conducted on characteristics such as hypolipidemic activity, kidney protection, cardiotonic activity, immune system improvement, antiarrhythmic, antiaging, and antineoplastic activities, etc.^[20]^. According to the information obtained from studies on fuziline, its protective and supportive importance against CVDs comes to the forefront.

In this study, the effectiveness of fuziline, which has an antioxidant effect, in preventing cardiac injury in mice exposed to cardiac injury by dobutamine, a positive inotropic agent *in vitro*, was histopathologically and biochemically investigated.

## METHODS

### Ethical Approval

Our study was conducted with the scientific committee approval of Harran University Animal Experiments Local Ethics Committee, dated 24/06/2021, session numbered 2021/005, decision 01-14.

### Determination of Study Groups and Cardiac Injury Model

Thirty-two adult male BALB/c mice with an average weight of 18-20 g were randomly divided into four groups (n=8) (Table 1). It was ensured that the mice were kept in cages, which allowed us to add fixed and transparent feed-water attachments, at a temperature of 22 ± 2°C, with a 50% relative humidity rate, in a 12-hour light and 12-hour dark environment. All mice were fed with standard mouse food and tap water under standard conditions. Group 1 (sham, n=8) was fed with standard mouse food and tap water for 15 days, and no procedure was performed. Group 2 (control, dobutamine, n=8) received 40 µg/mouse/day dobutamine intraperitoneally (IP) for 15 days. Group 3 (treatment 1, dobutamine + fuziline, n=8) received only 40 µg/mouse/day dobutamine for the first week IP. For the next week, fuziline was IP administered daily (3 mg/kg) in addition to dobutamine. Group 4 (treatment 2, fuziline, n=8) received only 3 mg/kg fuziline IP for 15 days. A mouse in the fuziline group died on the eighth day. Groups underwent electrocardiogram (ECG) on the eighth day. Mild sedation was applied to the dobutamine group before ECG. Dobutamine + fuziline group started to receive fuziline after injury determination. In total, this procedure lasted 15 days. Nutrition of all mice was discontinued eight hours before sacrifice. At the end of the experiment period (16^th^ day), all mice were sacrificed under deep anesthesia (ketamine 90 mg/kg and xylazine 10 mg/kg, IP). Blood and all tissues were collected.

**Table 1:** Study groups.

Groups	Weight	Gender	Genus	N=32 piece
Group 1 (sham)	18-20 g	Male	BALB/c mice	8
Group 2 (control, dobutamine)	18-20 g	Male	BALB/c mice	8
Group 3 (treatment 1, dobutamine + fuziline)	18-20 g	Male	BALB/c mice	8
Group 4 (treatment 2, fuziline)	18-20 g	Male	BALB/c mice	8

### Preparation of Dobutamine and Fuziline

Sigma brand 250 mg dobutamine was used to cause injury. Dobutamine (1.6 ml) was completed to 100 ml with saline; 0.1 ml was injected IP daily. Fuziline (Sigma) was obtained from the Turkish distributor of Interlab company. Fuziline (0.96 mg/kg) was dissolved in 1.6 ml dimethyl sulfoxide and administered to each mouse as 0.1 ml IP daily.

### Obtaining Blood Plasma and Study Method

Bloods collected from the heart and vena cava of the mice undergoing deep anesthesia were transferred to without anticoagulant yellow capped biochemistry tubes. Bloods were centrifuged at 4,000 rpm for 10 minutes. The plasma part was transferred to Eppendorf tubes and stored at -80°C until the day of the study. On the study day, the bloods were removed from -80°C and thawed at room temperature. Heart tissue samples from mice were placed in 10% formaldehyde for histopathological examination.

In our study, basal biochemical parameters were measured on certain devices as follows: alanine aminotransferase (ALT), urea, creatinine (Cr), sodium (Na), and potassium (K) were measured on the Atellica® Solution device, pro-brain natriuretic peptide (ProBNP) value was measured on AQT90 FLEX device, and troponin-I value was measured on Advia Centaur® XP Immunoassay System device. Siemens commercial kits were used for all basal biochemical parameters. Interleukin 1 beta (IL-1β) (CT LAB, Cat. No E0119Ra), NLR family, pyrin domain containing protein 3 (NLRP3) (CT Lab, Cat. No E1627Ra), 8-hydroxy-deoxyguanosine (8-OHDG) (CT LAB, Cat. No E0031Ra), gasdermin D (GSDMD) (CT LAB, Cat. No E2451Ra), and galectin 3 (GAL-3) (CT LAB, Cat. No E1533Ra) levels were examined through the enzyme-linked immunosorbent assay kit method. Total antioxidant status (TAS), total oxidant status (TOS), and oxidative stress index (OSI) values were measured in plasma. Histopathological examination of heart tissues of the mice was performed. Statistical analysis was conducted after all study data were obtained.

### Determination of Total Antioxidant Status

TAS of the samples was determined by Erel method^[21]^. The principle of TAS measurement is based on the reduction of the colored 2,2'-azino-bis (or ABTS) cationic radical by all antioxidant molecules in the sample. Trolox, a water-soluble analog of vitamin E, is used as a calibrator. TAS level was measured using Rel Assay Diagnostics® commercial kits. The results were expressed as mmol Trolox equivalent/L.

### Determination of Total Oxidant Status

TOS of the samples was determined by Erel method^[22]^. TOS level was measured using Rel Assay Diagnostics® commercial kits. The colorimetric method, which is based on the cumulative oxidation of the oxidant molecules in the samples to the ferrous ion, was used. The results were expressed as µmol H2O2 equivalent/L.

### Determination of Oxidative Stress Index

OSI calculation was carried out with Erel method^[21]^. OSI is expressed as the percentage of the rate of TOS levels to TAS levels. While calculating OSI, TAS levels are multiplied by 10, and units are equalized with TOS levels. The results were expressed as arbitrary units.


$$OSI=\frac{TOS,µmolH_{2}O_{2}\text{ equiv. }/L}{\text{ TAS, mmol Trolox equiv. }/L.\times 10}$$

### Histopathological Examination of Heart Tissue

Heart samples obtained from the mice were fixed by placing them in 10% formaldehyde. After appropriate samples were obtained, a four-hour tissue follow-up was performed on Leica BOND-MAX immunohistochemistry tissue tracking device, and then the samples were embedded in paraffin. Sections with 4 µm thickness were obtained from the heart of each mouse and stained with hematoxylin-eosin. In histopathological examination, the presence of necrosis, inflammation, and edema in the heart tissue was analyzed. The results were divided into groups as mild, moderate, and severe. In the quantitative histopathological examination of heart tissue, the whole region was scanned under the NiU microscope at 40× magnification in order to calculate the focal necrosis areas statistically. The mean necrosis area was calculated for each mouse. Two samples were obtained macroscopically while evaluating each heart tissue on an area of 0.23 mm^2^ at 40^×^ objective.

### Statistical Analysis

Normally distributed data were tested with Kolmogorov-Smirnov and Shapiro-Wilk tests. Independent samples *t*-test was used for normally distributed data of numerical variables, Mann-Whitney U test was used in the comparison of more than two independent groups for non-normally distributed data, while one-way analysis of variance and least significance difference multiple comparison tests were used for normally distributed characteristics, and Kruskal-Wallis test and all pairwise multiple comparison tests were used for non-normally distributed characteristics. As descriptive statistics, mean ± standard deviation was presented for numerical variables, while number and percentage values were presented for categorical variables. IBM Corp. Released 2016, IBM SPSS Statistics for Windows, version 24.0, Armonk, NY: IBM Corp. package program was used for statistical analysis, and *P*<0.05 was considered statistically significant.

## RESULTS

**Fig. 1:**
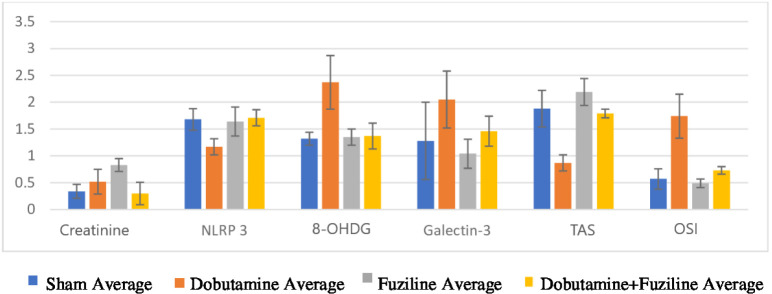
Mean measurements of creatinine, NLR family, pyrin domain containing protein 3 (NLRP3), 8-hydroxy-deoxyguanosine (8-OHDG), galectin 3, total antioxidant status (TAS), and oxidative stress index (OSI) between the groups.

Tables 2A and 2B show the statistical analysis of different parameters in sham, dobutamine, fuziline, and dobutamine + fuziline groups. According to this analysis, Cr, troponin-I, NLRP3, GSDMD, 8-OHDG, IL-1β, GAL-3, TOS, TAS, and OSI values were found to be statistically significant among the groups (*P*<0.05) (Figure 1). Pro-BNP, Na, K, ALT, and urea values were not statistically significant among the groups (*P*=0.051). While Cr value was measured as higher in the fuziline group (0.83±0.12 mg/dL), it was lower in the dobutamine group (0.52±0.23 mg/dL) and was considered statistically significant (*P*=0.001). When dobutamine + fuziline and fuziline groups were compared, troponin-I (pg/ml, *P*=0.025), NLRP3 (ng/ml, *P*=0.001), GSDMD (ng/L, *P*=0.001), 8-OHDG (ng/ml, *P*=0.001), IL-1β (pg/ml, *P*=0.001), and GAL-3 (ng/ml, *P*=0.004) were found to be statistically significant.

**Table 2a:** Statistical analysis of groups.

	Sham			Dobutamine			Fuziline			Dobutamine + Fuziline			
	Min.	Max.	SD	Min.	Max.	SD	Min.	Max.	SD	Min.	Max.	SD	*P*-value^a^
Pro-BNP (ng/l)	40.00	67.00	56.89 ± 8.71	49	69.9	60.93 ± 7.64	51.01	62.4	56.73 ± 4.67	59	69.99	65.37 ± 4,13	0.051
Urea (mg/dl)	11.96	26.90	19.72 ± 5.02	10.7	20.03	15.88 ± 2.99	10.7	25	16.99 ± 5.33	10	34.68	23.15 ± 9.21	0.109
Creatinine (mg/dl)	0.15	0.50	0.34 ± 0.13	0.16	0.9	0.52 ± 0.23	0.64	0.99	0.83 ± 0.12	0.1	0.61	0.34 ± 0.21	0.001^**^
ALT (U/l)	20.00	130.00	67.38 ± 35.64	10	65	40.63 ± 22.22	13	85	45.57 ± 30.46	17	99	46.25 ± 27.23	0.291
Sodium (mmol/l)	159.00	164.00	160.88 ± 1.81	159	163	161 ± 1.31	160	163	161.71 ± 1.11	159	168	162.38 ± 2.62	0.345
Potassium (mmol/l)	3.40	4.10	3.74 ± 0.26	3.5	4.1	3.75 ± 0.17	3.3	4.4	3.71 ± 0.36	3.2	3.9	3.65 ± 0.23	0.872
Troponin-I (pg/ml)	2276.85	9919.89	5046 ± 2306	2424.7	13518.08	6775 ± 4182	3211.77	5970.83	4435 ± 1000	4365.06	13677.76	8967 ± 3174	0.025^*^

a Analysis of variance test,

* *P*<0.05,

** *P*<0.001

ALT=alanine aminotransferase; ProBNP=pro-brain natriuretic peptide; SD=standard deviation

**Table 2b:** Statistical analysis of groups.

	Sham			Dobutamine			Fuziline			Dobutamine + Fuziline			
	Min.	Max.	SD	Min.	Max.	SD	Min.	Max.	SD	Min.	Max.	SD	*P*-value^a^
NLRP3 (ng/ml)	1.43	2.04	1.68 ± 0.2	0.973	1.422	1.17 ± 0.15	1.397	2.102	1.64 ± 0.27	1.44	1.908	1.71 ± 0.15	0.001^**^
GSDMD (ng/l)	256.30	428.58	341.14 ± 60.01	503.362	750.2	588.91 ± 80.22	140.492	153.591	145.27 ± 5.36	140.201	599.628	431.94 ± 145.05	0.001^**^
8-OHDG (ng/ml)	1.15	1.54	1.32 ± 0.12	1.238	2.952	2.37 ± 0.5	1.149	1.552	1.35 ± 0.15	1.03	1.813	1.37 ± 0.24	0.001^**^
IL-1β (pg/ml)	422.03	824.23	569.32 ± 124.79	817.291	1.388.563	1012.32 ± 187.83	431.356	823.101	559.83 ± 133.21	428.42	850.3	713.23 ± 137.24	0.001^**^
GAL-3 (ng/ml)	0.75	2.94	1.28 ± 0.72	1.411	2.948	2.05 ± 0.53	0.823	1.556	1.04 ± 0.27	0.886	1.715	1.46 ± 0.28	0.004^*^
TOS (µmol H2O2 equivalent/l)	7.20	13.10	10.1 ± 2.08	11.3	16.2	14.6 ± 1.66	9.52	12.1	10.67 ± 0.87	11.2	14.2	13.06 ± 1.01	0.001^**^
TAS (mmol Trolox equivalent/l)	1.20	2.30	1.88 ± 0.34	0.66	1.09	0.87 ± 0.15	1.96	2.6	2.19 ± 0.25	1.69	1.92	1.79 ± 0.08	0.001^**^
OSI (AU)	0.35	0.88	0.57 ± 0.19	1.11	2.3	1.74 ± 0.41	0.38	0.58	0.49 ± 0.08	0.59	0.82	0.73 ± 0.07	0.001^**^

a Analysis of variance test,

* *P*<0.05,

** *P*<0.001

8-OHDG=8-hydroxy-deoxyguanosine; AU=arbitrary units; GAL-3=galectin 3; GSDMD=gasdermin D; IL-1β=interleukin 1 beta; NLRP3=NLR family, pyrin domain containing protein 3; OSI=oxidative stress index; SD=standard deviation; TAS=total antioxidant status; TOS=total oxidant status

TOS value was at the highest level in the dobutamine group (14.6±1.66 µmol H2O2 equivalent/L). In the dobutamine + fuziline group, the TOS value was found to be low (13.06±1.01 µmol H2O2 equivalent/ L) (*P*=0.001). TAS value was at the highest level in the fuziline group (2.19±0.25 mmol Trolox equivalent/L), lower in the dobutamine + fuziline group (1.79±0.08 mmol Trolox equivalent/L), and at the lowest level in the dobutamine group (0.87±0.15 mmol Trolox equivalent/L) (*P*=0.001). OSI levels were found to be statistically significant in the groups in parallel with TAS and TOS values (*P*=0.001).

Correlation analyses are presented in Table 3. According to this analysis, there is a negative and highly significant relationship between GSDMD and TAS (r=-0.748, *P*=0.001). There is a highly positive significant relationship between 8-OHDG and IL-1β values (r=0.722, *P*=0.001) and between 8-OHDG and OSI values (r=0.773, *P*=0.001). There is a highly negative significant relationship between 8-OHDG and TAS values (r=-0.759, *P*=0.001). There is a highly positive relationship between IL-1β and GAL-3 values (r=0.865, *P*=0.001), between IL-1β and TOS values (r=0.735, *P*=0.001), and between IL-1β and OSI values (r=0.796, *P*=0.001), and there is a negative and highly significant relationship between IL-1β and TAS values (r=-0.739, *P*=0.001). There is a highly positive and significant relationship between GAL-3 and TOS (r=0.689, *P*=0.001) and between GAL-3 and OSI values (r=0.676, *P*=0.001). There is a highly positive and significant relationship between TOS and OSI values (r=0.797, *P*=0.001). And there is a highly negative and significant relationship between TAS and OSI values (r=-0.928, *P*=0.001).

**Table 3:** Correlation analysis.

		NLRP3	GSDMD	8-OHDG	IL-1β	GAL-3	TOS	TAS	OSI
NLRP3 (ng/ml)	r	1.00	-.503^**^	-.607^**^	-0.33	-0.18	-.367^*^	.663^**^	-.656^**^
	*P*-value		0.001	0.001	0.07	0.34	0.04	0.001	0.001
GSDMD (ng/L)	r	-.503^**^	1.00	.510^**^	.634^**^	.506^**^	.609^**^	-.748^**^	.669^**^
	*P*-value	0.001		0.001	0.001	0.001	0.001	0.001	0.001
8-OHDG (ng/ml)	r	-.607^**^	.510^**^	1.00	.722^**^	.559^**^	.578^**^	-.759^**^	.773^**^
	*P*-value	0.001	0.001		0.001	0.001	0.001	0.001	0.001
IL-1β (pg/ml)	r	-0.33	.634^**^	.722^**^	1.00	.865^**^	.735^**^	-.739^**^	.796^**^
	*P*-value	0.07	0.001	0.001		0.001	0.001	0.001	0.001
GAL-3 (ng/ml)	r	-0.18	.506^**^	.559^**^	.865^**^	1.00	.689^**^	-.590^**^	.676^**^
	*P*-value	0.34	0.001	0.001	0.001		0.001	0.001	0.001
TOS (µmol H2O2 equivalent/L)	r	-.367^*^	.609^**^	.578^**^	.735^**^	.689^**^	1.00	-.698^**^	.797^**^
	*P*-value	0.04	0.001	0.001	0.001	0.001		0.001	0.001
TAS (mmol Trolox equivalent/L)	r	.663^**^	-.748^**^	-.759^**^	-.739^**^	-.590^**^	-.698^**^	1.00	-.928^**^
	*P*-value	0.001	0.001	0.001	0.001	0.001	0.001		0.001
OSI (AU)	r	-.656^**^	.669^**^	.773^**^	.796^**^	.676^**^	.797^**^	-.928^**^	1.00
	*P*-value	0.001	0.001	0.001	0.001	0.001	0.001	0.001	

* *P*<0.05,

** *P*<0.001

8-OHDG=8-hydroxy-deoxyguanosine; AU=arbitrary units; GAL-3=galectin 3; GSDMD=gasdermin D; IL-1β=interleukin 1 beta; NLRP3=NLR family, pyrin domain containing protein 3; OSI=oxidative stress index; TAS=total antioxidant status; TOS=total oxidant status

### Histopathological Examination Results of Heart Tissue

**Fig. 2:**
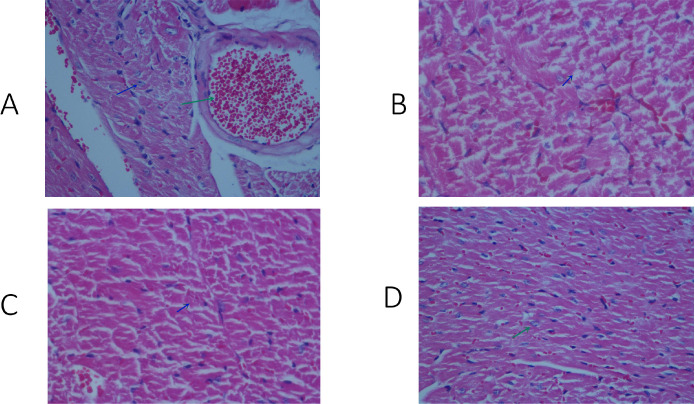
Histopathological examination of heart tissue belonging to the groups. A) Sham group, B) dobutamine group, C) dobutamine + fuziline group, and D) fuziline group. Green arrows indicate normal myocyte cells. Blue arrows indicate myocyte necrosis.

**Fig. 3:**
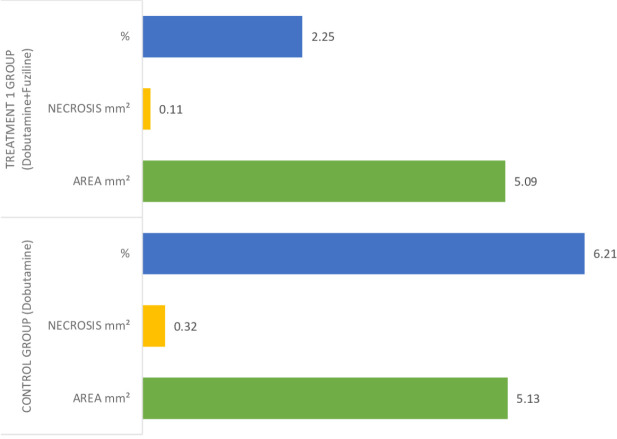
Comparison of necrosis areas of dobutamine and dobutamine + fuziline groups.

When the heart muscles of 15 mice in the sham and fuziline groups were examined, it was observed that the regular histological structure was preserved. In the histopathological examination of the dobutamine group, focal necrosis areas were partly observed in the heart muscle tissues of all mice. The rate of injury in the heart muscle was evaluated as moderate. In the histopathological examination of the dobutamine + fuziline group, the presence of focal necrosis areas was observed; however, these areas were more limited compared to the dobutamine group. The rate of injury to the heart muscle was evaluated as mild (Figure 2). Necrosis areas were calculated for each pathological sample according to the tissue surface area in the cardiac muscle sections obtained histopathologically (mm^2^). The median value of necrosis in the dobutamine group was 6.21% (Table 4), while it was 2.25% in the dobutamine + fuziline group (Table 5) (Figure 3).

**Table 4:** Dobutamine group (control) cardiac necrosis areas.

	Dobutamine group (control)		
	Area (mm^2^)	Necrosis (mm^2^)	%
1	4.83	0.34	7.00
2	4.60	0.28	6.00
3	5.29	0.26	5.00
4	5.06	0.40	8.00
5	5.52	0.39	7.00
6	5.52	0.30	5.50
7	5.06	0.25	5.00
Median (mm^2^)	5.13	0.32	6.21

**Table 5:** Dobutamine + fuziline group (treatment 1) cardiac necrosis areas.

	Dobutamine + fuziline group (treatment 1)		
	Area (mm^2^)	Necrosis (mm^2^)	%
1	4.60	0.05	1.00
2	5.29	0.13	2.50
3	5.52	0.15	2.75
4	5.06	0.08	1.50
5	5.29	0.16	3.00
6	4.83	0.10	2.00
7	5.06	0.14	2.75
Median (mm^2^)	5.09	0.11	2.25

## DISCUSSION

Pyroptosis is defined as programmed cell death due to inflammation^[23]^ and is initiated due to various pathological conditions including myocardial infarction (MI)^[24]^, inflammation, oxidative stress, etc.^[25]^. Pyroptosis involves cell lysis and release of intracellular proinflammatory content through caspase-1 enzyme^[26]^. Mezzaroma et al.^[24]^ explained that MI activates the multiple protein inflammatory compound consisting of apoptosis-associated speck-like protein containing caspase-1, NLRP3, and a caspase recruitment domain and causes pyroptosis to emerge in cardiomyocytes. The inflammatory activity of NLRP3 leads to the emergence of immune inflammatory reactions. It is also involved in cardiovascular ischemia-reperfusion injury, HF, and atherosclerosis^[27]^. A surgical operation or transverse aortic constriction was performed in mice by Li et al.^[28]^. According to the results obtained from their study, NLRP3 levels were observed to increase in mice undergoing the procedure, and this situation caused cardiomyocyte hypertrophy, myocardial fibrosis, and cardiac dysfunction. Sandanger et al.^[29]^ created MI in rats and mice through coronary artery ligation. Following MI, it was explained that IL-1β, interleukin 18 (IL-18), and NLRP3 levels increased in heart tissue, heart functions were better in mice with NLRP3 deficiency, and the MI area was at a lower level. Moreover, NLRP3 regulates the formation and circulation of proinflammatory cytokines such as IL-18 and IL-1β^[30]^. Inactivation of IL-1β has positive results in terms of CVD^[31]^ because it has a critical function at the onset of vascular inflammatory disorders and atherogenesis^[32]^. Du et al.^[33]^ randomly divided 32 apolipoprotein E-/-mice into two groups. IL-1β, IL-18, and NLRP3 levels were examined between the groups by subjecting one group to filtered air and the other group to main air-polluting fine particulate matter (≤ 2.5 µm; particulate matter 2.5 [PM2.5]). In mice exposed to PM2.5, NLRP3 inflammatory activity and IL-1β and IL-18 levels were found to be increased, and atherosclerotic plaques increased in the aorta.

Pyroptosis-associated extracellular signals divide and activate caspase-1, -4, -5, and -11 after activating inflammasomes such as NLRP3. Following these activities, caspase-1 is activated, and gasdermin is activated by splitting into C-terminal and N-terminal^[34]^. Due to the perforation and lipophilicity characteristics of the N-terminal, all types of gasdermin (GSDMA, GSDMB, GSDMC, GSDMD, and GSDME/DFNA5), except for DFNB59, bind to C-terminal and causes inhibited pyroptosis. N-terminal fragment of GSDMD plays a role in the release of pro-inflammatory agents (IL-1β, IL-18, etc.) and cell swelling by forming pores in the cell membrane^[35]^. GSDMD was the first gasdermin discovered to be associated with pyroptosis^[34]^. GSDMD is decomposed through inflammatory caspase-1, -4, -5, and -11 or non-inflammatory caspase-8 to induce pyroptosis^[36]^. While caspase-1/GSDMD or caspase-3/GSDME plays a role in the activity of pyroptosis, the activity of other types of gasdermin has not been fully explained^[37]^. Yang et al.^[38]^ created a diabetic model with streptozotocin in mice. They explained that a critical increase was observed in GSDMD-N, IL-1β, NLRP3, and caspase-1 levels in the heart tissue of these mice. In their *in vitro* study, Dargani et al.^[39]^ intervened H9c2 cells with doxorubicin to clarify whether doxorubicin triggered pyroptosis. It was observed that pyroptosis was initiated with the detection of NLRP3. In addition, this situation was confirmed with pyroptosis markers such as GSDMD, caspase-1, and IL-1β.

8-OHDG is an oxidized structure of guanosine involved in disorders such as atherosclerosis, diabetes, and cancer and is an indicator of oxidative deoxyribonucleic acid (DNA) damage^[40]^. Di Minno et al.^[41]^ compared patients with HF and control group patients and explained that the level of 8-OHDG was higher in patients with HF. Wang et al.^[42]^ initiated sepsis with cecal ligation and puncture in mice. Following examinations revealed an increase in 8-OHDG and inflammatory factor levels.

GAL-3 is a new biomarker of CVDs. It has an important role in determining the path that oxidative stress and inflammatory response will follow. Furthermore, it is effective in the development of atherosclerosis with its effects such as lipid endocytosis, endothelial dysfunction, etc.^[43]^. In their study, De Boer et al.^[44]^ revealed that GAL-3 was associated with both gender and age factors and CVD risk factors. Van der Velde et al.^[45]^ measured GAL-3 level in 5,958 participants and conducted a mean 8.3-year follow-up. As a result of this study, they explained that a constantly high GAL-3 level may indicate the onset of HF.

Many damage mechanisms are related to the formation of cardiac injury. Beta agonists play a leading role in many of these methods. In their study, Fan et al.^[46]^ examined aspartate aminotransferase, lactate dehydrogenase, creatine kinase, and creatine kinase-myocardial band levels in plasma in order to determine the myocardial injury they created with ISO in male Sprague Dawley rats and found that these parameters were higher in the ISO group. Similarly, Anderson et al.^[47]^ examined the structure and function of the heart after continuing dobutamine (40 µg/mouse/day) to female mice for seven days. They found that cardiac wet weight increased by 24% following the dobutamine dose administered on the 7^th^ day, the functionality of the heart decreased, and cardiac fibrosis increased significantly. In our study, a model similar to these mechanisms was selected. Following the administration of 40 µg/mouse/day of dobutamine to the mice in the dobutamine group for 15 days, the cardiac injury was formed by an increase in oxidative stress parameters and troponin-I.

### Limitations

There are several limitations in this study. First, the groups were not formed depending on different doses of dobutamine. And another is that the content of fuziline consists of different molecules and different dosage options were not applied, similar to dobutamine.

## CONCLUSION

In our study, the levels of GSDMD and IL-1β (pyroptosis markers), 8-OHDG (oxidative DNA damage marker), and GAL-3 (oxidative stress marker) decreased with the administration of fuziline. This result shows that fuziline can be seriously protective against cardiac injury caused by various mechanisms and prevent necrosis of myocytes. Future comprehensive studies will more distinctively reveal the effectiveness of fuziline in preventing pyroptosis and oxidative stress.

**Table:** 

Authors’ Roles & Responsibilities	
YH	Substantial contributions to the conception and design of the work; and the acquisition, analysis and interpretation of data for the work; drafting the work and revising it critically for important intellectual content; final approval of the version to be published
MSA	Substantial contributions to the acquisition, analysis and interpretation of data for the work; drafting the work and revising it; final approval of the version to be published
EKE	Substantial contributions to the acquisition, analysis and interpretation of data for the work; drafting the work and revising it; final approval of the version to be published
NK	Substantial contributions to the acquisition, analysis and interpretation of data for the work; drafting the work and revising it; final approval of the version to be published
İK	Substantial contributions to the conception and design of the work; revising it critically for important intellectual content; final approval of the version to be published
MEG	Substantial contributions to the conception and design of the work; revising it critically for important intellectual content; final approval of the version to be published
ET	Substantial contributions to the conception and design of the work; revising it critically for important intellectual content; final approval of the version to be published
RD	Substantial contributions to the conception and design of the work; revising it critically for important intellectual content; final approval of the version to be published
KE	Substantial contributions to the conception and design of the work; revising it critically for important intellectual content; final approval of the version to be published
YÇ	Substantial contributions to the conception and design of the work; revising it critically for important intellectual content; final approval of the version to be published
MP	Substantial contributions to the conception and design of the work; revising it critically for important intellectual content; final approval of the version to be published

## References

[1] Tanrıverdi B, Tetik ŞS (2017). Aterosklerozun Patofizyolojisi ve Risk
Faktörleri. Mar Pharmac J.

[2] Sarrafzadegan N, Gotay C. (2015). CVD prevention in 2014: advances in the prevention of
cardiovascular disease. Nat Rev Cardiol.

[3] Yancy CW, Jessup M, Bozkurt B, Butler J, Casey DE Jr, Drazner MH (2013). Fonarow GC, 2013 ACCF/AHA guideline for the management of heart
failure: a report of the American college of cardiology foundation/American
heart association task force on practice guidelines. J Am Coll Cardiol.

[4] Hensyl WR (1990). Stedman’s medical Dictionary.

[5] Rivers E, Nguyen B, Havstad S, Ressler J, Muzzin A, Knoblich B (2001). Early goal-directed therapy in the treatment of severe sepsis and
septic shock. N Engl J Med.

[6] Abraham WT, Adams KF, Fonarow GC, Costanzo MR, Berkowitz RL, LeJemtel TH (2005). In-hospital mortality in patients with acute decompensated heart
failure requiring intravenous vasoactive medications: an analysis from the
acute decompensated heart failure national registry (ADHERE). J Am Coll Cardiol.

[7] Halliwell B (2007). Biochemistry of oxidative stress. Biochem Soc Trans.

[8] Valko M, Leibfritz D, Moncol J, Cronin MT, Mazur M, Telser J (2007). Free radicals and antioxidants in normal physiological functions
and human disease. Int J Biochem Cell Biol.

[9] Taysi S, Tascan AS, Ugur MG, Demir M (2019). Radicals, oxidative/nitrosative stress and
preeclampsia. Mini Rev Med Chem.

[10] Bruni A, Bornstein S, Linkermann A, Shapiro AMJ (2018). Regulated cell death seen through the lens of islet
transplantation. Cell Transplant.

[11] Köksal E, Gülçin İ (2008). Antioxidant Activity of Cauliflower (Brassica oleraceae
L.). Turk J Agric For.

[12] Erdemli ME, Aksungur Z, Gul M, Yigitcan B, Bag HG, Altinoz E (2019). The effects of acrylamide and vitamin E on kidneys in pregnancy:
an experimental study. J Matern Fetal Neonatal Med.

[13] Kocaman G, Altinoz E, Erdemli ME, Gul M, Erdemli Z, Zayman E (2021). Crocin attenuates oxidative and inflammatory stress-related
periodontitis in cardiac tissues in rats. Adv Clin Exp Med.

[14] Erdemli ME, Yigitcan B, Gul M, Bag HG, Gul S, Aksungur Z (2018). Thymoquinone is protective against
2,3,7,8-tetrachlorodibenzo-p-dioxin induced hepatotoxicity. Biotech Histochem.

[15] Priscilla DH, Prince PS (2009). Cardioprotective effect of gallic acid on cardiac troponin-T,
cardiac marker enzymes, lipid peroxidation products and antioxidants in
experimentally induced myocardial infarction in Wistar rats. Chem Biol Interact.

[16] Rodríguez-Pérez C, Segura-Carretero A, Del Mar Contreras M (2019). Phenolic compounds as natural and multifunctional anti-obesity
agents: a review. Crit Rev Food Sci Nutr.

[17] Baek SH, Cao L, Jeong JS, Kim HR, Nam J T, Lee GS (2021). The Comparison of Total Phenolics, Total Antioxidant, and
Anti-Tyrosinase Activities of Korean Sargassum Species. J Food.

[18] Chinese Pharmacopoeia Commission (2010). Pharmacopoeia of the People’s Republic of China.

[19] Zhou G, Tang L, Zhou X, Wang T, Kou Z, Wang Z (2015). A review on phytochemistry and pharmacological activities of the
processed lateral root of aconitum carmichaelii Debeaux. J Ethnopharmacol.

[20] Ren MY, Yu QT, Shi CY, Luo JB (2017). Anticancer activities of C18-, C19-, C20-, and bis-diterpenoid
alkaloids derived from genus aconitum. Molecules.

[21] Erel O (2004). A novel automated direct measurement method for total antioxidant
capacity using a new generation, more stable ABTS radical
cation. Clin Biochem.

[22] Erel O (2005). A new automated colorimetric method for measuring total oxidant
status. Clin Biochem.

[23] Hu Q, Zhang T, Yi L, Zhou X, Mi M (2018). Dihydromyricetin inhibits NLRP3 inflammasome-dependent pyroptosis
by activating the Nrf2 signaling pathway in vascular endothelial
cells. Biofactors.

[24] Mezzaroma E, Toldo S, Farkas D, Seropian IM, Van Tassell BW, Salloum FN (2011). The inflammasome promotes adverse cardiac remodeling following
acute myocardial infarction in the mouse. Proc Natl Acad Sci U S A.

[25] Reisetter AC, Stebounova LV, Baltrusaitis J, Powers L, Gupta A, Grassian VH (2011). Induction of inflammasome-dependent pyroptosis by carbon black
nanoparticles. J Biol Chem.

[26] Fink SL, Cookson BT (2005). Apoptosis, pyroptosis, and necrosis: mechanistic description of
dead and dying eukaryotic cells. Infect Immun.

[27] Wang Y, Liu X, Shi H, Yu Y, Yu Y, Li M (2020). NLRP3 inflammasome, an immune-inflammatory target in pathogenesis
and treatment of cardiovascular diseases. Clin Transl Med.

[28] Li R, Lu K, Wang Y, Chen M, Zhang F, Shen H (2017). Triptolide attenuates pressure overload-induced myocardial
remodeling in mice via the inhibition of NLRP3 inflammasome
expression. Biochem Biophys Res Commun.

[29] Sandanger Ø, Ranheim T, Vinge LE, Bliksøen M, Alfsnes K, Finsen AV (2013). The NLRP3 inflammasome is up-regulated in cardiac fibroblasts and
mediates myocardial ischaemia-reperfusion injury. Cardiovasc Res.

[30] Shi J, Gao W, Shao F (2017). Pyroptosis: gasdermin-mediated programmed necrotic cell
death. Trends Biochem Sci.

[31] Ridker PM, Everett BM, Thuren T, MacFadyen JG, Chang WH, Ballantyne C (2017). Antiinflammatory therapy with canakinumab for atherosclerotic
disease. N Engl J Med.

[32] Eun SY, Ko YS, Park SW, Chang KC, Kim HJ (2015). IL-1β enhances vascular smooth muscle cell proliferation
and migration via P2Y2 receptor-mediated RAGE expression and HMGB1
release. Vascul Pharmacol.

[33] Du X, Jiang S, Zeng X, Zhang J, Pan K, Zhou J (2018). Air pollution is associated with the development of
atherosclerosis via the cooperation of CD36 and NLRP3 inflammasome in
ApoE-/- mice. Toxicol Lett.

[34] Shi J, Zhao Y, Wang K, Shi X, Wang Y, Huang H (2015). Cleavage of GSDMD by inflammatory caspases determines pyroptotic
cell death. Nature.

[35] Ding J, Wang K, Liu W, She Y, Sun Q, Shi J (2016). Pore-forming activity and structural autoinhibition of the
gasdermin family. Nature.

[36] Kambara H, Liu F, Zhang X, Liu P, Bajrami B, Teng Y (2018). Gasdermin D exerts anti-inflammatory effects by promoting
neutrophil death. Cell Rep.

[37] Wang L, Qin X, Liang J, Ge P (2021). Induction of pyroptosis: a promising strategy for cancer
treatment. Front Oncol.

[38] Yang F, Qin Y, Lv J, Wang Y, Che H, Chen X (2018). Silencing long non-coding RNA Kcnq1ot1 alleviates pyroptosis and
fibrosis in diabetic cardiomyopathy. Cell Death Dis.

[39] Tavakoli Dargani Z, Singla DK (2019). Embryonic stem cell-derived exosomes inhibit doxorubicin-induced
TLR4-NLRP3-mediated cell death-pyroptosis. Am J Physiol Heart Circ Physiol.

[40] Gao Y, Wang P, Wang Z, Han L, Li J, Tian C (2019). Serum 8-Hydroxy-2'-Deoxyguanosine level as a potential biomarker
of oxidative DNA damage induced by ionizing radiation in human peripheral
blood. Dose Response.

[41] Di Minno A, Turnu L, Porro B, Squellerio I, Cavalca V, Tremoli E (2017). 8-Hydroxy-2-deoxyguanosine levels and heart failure: a systematic
review and meta-analysis of the literature. Nutr Metab Cardiovasc Dis.

[42] Wang C, Yuan W, Hu A, Lin J, Xia Z, Yang CF (2020). Dexmedetomidine alleviated sepsis-induced myocardial ferroptosis
and septic heart injury. Mol Med Rep.

[43] Srivatsan V, George M, Shanmugam E (2015). Utility of galectin-3 as a prognostic biomarker in heart failure:
where do we stand?. Eur J Prev Cardiol.

[44] de Boer RA, van Veldhuisen DJ, Gansevoort RT, Muller Kobold AC, van Gilst WH, Hillege HL (2012). The fibrosis marker galectin-3 and outcome in the general
population. J Intern Med.

[45] van der Velde AR, Meijers WC, Ho JE, Brouwers FP, Rienstra M, Bakker SJ (2016). Serial galectin-3 and future cardiovascular disease in the
general population. Heart.

[46] Fan CL, Yao ZH, Ye MN, Fu LL, Zhu GN, Dai Y (2020). Fuziline alleviates isoproterenol-induced myocardial injury by
inhibiting ROS-triggered endoplasmic reticulum stress via
PERK/eIF2α/ATF4/Chop pathway. J Cell Mol Med.

[47] Anderson M, Moore D, Larson D (2008). Comparison of isoproterenol and dobutamine in the induction of
cardiac hypertrophy and fibrosis. Perfusion.

